# Insecticidal and Antifeedant Activities of Malagasy Medicinal Plant (*Cinnamosma* sp.) Extracts and Drimane-Type Sesquiterpenes against *Aedes aegypti* Mosquitoes

**DOI:** 10.3390/insects10110373

**Published:** 2019-10-25

**Authors:** Edna Alfaro Inocente, Bao Nguyen, Preston K. Manwill, Annecie Benatrehina, Eliningaya Kweka, Sijin Wu, Xiaolin Cheng, L. Harinantenaina Rakotondraibe, Peter M. Piermarini

**Affiliations:** 1 Department of Entomology, The Ohio State University, Ohio Agricultural Research and Development Center, Wooster, OH 44691, USA; alfaroinocente.1@buckeyemail.osu.edu (E.A.I.); bnguyen20@wooster.edu (B.N.); 2 Departments of Medicinal Chemistry and Pharmacognosy, The Ohio State University, Columbus, OH 43210, USA; manwill.1@buckeyemail.osu.edu (P.K.M.); Benatrehina.1@osu.edu (A.B.); wu.3857@osu.edu (S.W.); cheng.1302@osu.edu (X.C.); 3 Center for Applied Plant Sciences, The Ohio State University, Columbus, OH 43210, USA; 4 Department of Medical Parasitology, School of Medicine, Catholic University of Health and Allied Sciences-Bugando, Mwanza P.O. Box 1464, Tanzania; pat.kweka@gmail.com; 5 Division of Livestock and Human Disease Vector Control, Tropical Pesticides Research Institute, Arusha P.O. Box 3024, Tanzania

**Keywords:** *Aedes aegypti*, *Cinnamosma*, *Warburgia*, insecticide, antifeedant

## Abstract

The overuse of insecticides with limited modes of action has led to resistance in mosquito vectors. Thus, insecticides with novel modes of action are needed. Secondary metabolites in Madagascan plants of the genus *Cinnamosma* (Canellaceae) are commonly used in traditional remedies and known to elicit antifeedant and toxic effects in insect pests. Here we test the hypothesis that extracts of *Cinnamosma* sp. enriched in drimane sesquiterpenes are toxic and/or antifeedant to the yellow fever mosquito *Aedes aegypti*. We show that the bark and root extracts, which contain a higher abundance of drimane sesquiterpenes compared to leaves, were the most efficacious. Screening isolated compounds revealed cinnamodial to be the primary driver of adulticidal activity, whereas cinnamodial, polygodial, cinnafragrin A, and capsicodendrin contributed to the larvicidal activity. Moreover, an abundant lactone (cinnamosmolide) in the root extract synergized the larvicidal effects of cinnamodial. The antifeedant activity of the extracts was primarily contributed to cinnamodial, polygodial, and cinnamolide. Parallel experiments with warburganal isolated from *Warburgia ugandensis* (Canellaceae) revealed that aldehydes are critical for—and a hydroxyl modulates—insecticidal activity. Our results indicate that plant drimane sesquiterpenes provide valuable chemical platforms for developing insecticides and repellents to control mosquito vectors.

## 1. Introduction

The mosquito *Aedes aegypti* (Linnaeus in Hasselquist, 1762) (Diptera: Culicidae) inhabits tropical and subtropical habitats worldwide. It is the predominant vector of numerous medically important arboviruses, such as dengue fever, chikungunya, yellow fever, and Zika, which infect hundreds of millions of people each year. The traditional approaches to combating *Ae. aegypti* using synthetic pesticides, such as pyrethroids and dichlorodiphenyltrichloroethane (DDT), have potential side effects on both the environment and human health, and their overuse has contributed to the development of insecticide resistance in the mosquito [1,2,3,4].

In recent years, interest in plant-based insecticides has grown because of this development of resistance and off-target effects of synthetic pesticides [5,6,7,8]. Secondary metabolites of plants have been shaped by millions of years of natural selection to protect plants against herbivores, including insects. Thus, they represent a potentially exciting library of bioactive compounds to screen for the discovery of novel insecticides and repellents. Moreover, they offer alternatives to synthetic insecticides and repellents that are potentially safer for the environment and human health [8,9,10,11,12]. Examples of insecticides or repellents developed from natural plant products include azadirachtin [13], citronellal [14], geraniol [15], *p*-menthane-3,8-diol [16],pyrethrum [17,18], nicotine, and ryanodine [19].

Recent studies by our group and others have demonstrated that plants of the genus *Cinnamosma* (Baillon) (Canellaceae) (*C. fragrans*, *C. macrocarpa,* and *C. madagascariensis*) are a potential source of novel insecticides and repellents for controlling mosquitoes [20,21]. These plants were first described by Henri Baillon as small trees with a pleasant smell that were widely distributed in the northern and eastern parts of Madagascar. The decoctions of the bark and root bark have a distinct pepper-like taste, and essential oils obtained from their different parts have been used by Malagasy people for generations as a traditional medicine for malaria, respiratory problems, muscle aches, and gastrointestinal infections [22,23,24,25,26]. The use of *Cinnamosma* spp. as remedies for a wide range of ailments suggests that plants from this genus produce diverse and highly bioactive phytochemicals [25]. Moreover, the common uses of the plants in traditional medicine suggest a potentially safe toxicological profile of the bioactive molecules in humans.

**Figure 1:**
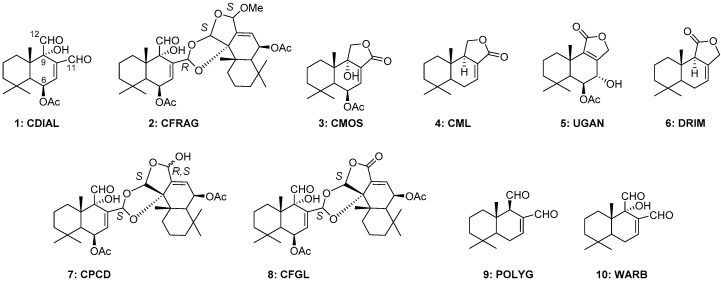
Structures of isolated compounds. Carbons referred to in the text are numbered in cinnamodial (CDIAL). CFRAG = cinnafragrin A; CMOS = cinnamosmolide; CML = cinnamolide; UGAN = ugandensolide; DRIM = drimenin; CPCD = capsicodendrin; CFGL = cinnafragrolide; POLYG = polygodial; WARB = warburganal.

We previously demonstrated that the dichloromethane extract of the bark of *C. fragrans*, which is enriched with pungent drimane sesquiterpenes, was insecticidal, antifeedant, and repellent to mosquitoes [20]. The most abundant drimane sesquiterpene in the extract, a dialdehyde known as cinnamodial (CDIAL, **1**; Figure 1), was insecticidal against larval and adult female mosquitoes (*Ae. aegypti*, *Anopheles gambiae*, *Culex pipiens*). Notably, **1** exhibited similar toxic potency against pyrethroid-susceptible and pyrethroid-resistant strains of *Ae. aegypti*, suggesting a novel mode of action from pyrethroids. Our studies also revealed that **1** was an agonist of mosquito TRPA1 channels, which was the likely mode of antifeedant and repellent actions. However, TRPA1 modulation was not necessary for CDIAL’s insecticidal activity, suggesting distinct modes of insecticidal and antifeedant/repellent activity. We also tested two other drimane sesquiterpenes in the bark extract: 1) Cinnafragrin A (CFRAG, **2**), a dimeric derivative of **1** containing a single aldehyde; and 2) cinnamosmolide (CMOS, **3**), a lactone-bearing derivative of **1** (Figure 1). Compounds **2** and **3** showed relatively weak bioactivities against mosquitoes compared to **1** [20].

Due to the promising bioactivities of the dichloromethane extract of *C. fragrans* bark and **1**, here we evaluated the relative insecticidal and antifeedant activities of dichloromethane extracts from other parts of *Cinnamosma* plants (roots, leaves), which have not previously been determined. Furthermore, *Cinnamosma* spp. have been reported to produce numerous other CDIAL-like sesquiterpenes (Figure 1), including: lactone-bearing compounds, such as cinnamolide (CML, **4**), ugandensolide (UGAN, **5**), and drimenin (DRIM, **6**); dimers, such as capsicodendrin (CPCD, **7**) and the oxidation derivative cinnafragrolide (CFGL, **8**); and another dialdehyde, polygodial (POLYG, **9**) [25,27]. Here we evaluate the insecticidal and antifeedant activities of these molecules, which have never been determined against mosquitoes.

Furthermore, to advance the structure–activity relationship (SAR) of **1**, we evaluated the insecticidal and antifeedant activity of warburganal (WARB, **10**; Figure 1), a drimane sesquiterpene dialdehyde isolated from the bark of a South-eastern African medicinal plant (*Warburgia ugandensis*, Canellaceae) closely related to *Cinnamosma* spp. Compound **10** has previously been shown to possess potent antifeedant activity against lepidopteran pests [28,29] but has not been tested in mosquitoes.

## 2. Materials and Methods

### 2.1. Plant Material and Isolation of Chemicals

Bark extract was prepared from *Cinnamosma fragrans* purchased in the market of traditional medicine in Analakely/Antananarivo (Madagascar), while the leaf and root extracts were prepared from *Cinnamosma madagascariensis* collected in the Mangoro region, Madagascar. The air-dried stem bark, roots, or leaves, were separated and pulverized, and the resulting powder of each sample was macerated with dichloromethane for 2 days at room temperature. The extracts were filtered and concentrated in vacuo to yield stem bark, root, and leaves extracts. Isolation and structure elucidation of compounds **1**–**9** have been previously described [27,30,31]. In brief, the extract was filtered and concentrated in vacuo to yield a yellow-brown oily residue. The residue was divided into 35 fractions (F01-35) using column chromatography over silica gel, eluting with a gradient system of hexanes–EtOAc (from 4:1 to 0:1). Ugandensolide (**5**) was crystallized by slow evaporation of F26, and the crystals rinsed with cold hexanes–EtOAc (1:1) to yield orange crystals. The structure of **5** was confirmed by spectral comparison to those previously published [32,33]. CDIAL (**1**) was recrystallized from fraction 15 using hexanes–EtOAc (1:1) as colorless crystals. The fractions containing CPCD (**7**) (F22-23) were rinsed with ethyl acetate, and the supernatant removed to obtain **7** as a white amorphous solid.

Warburganal (**10**) was isolated from the bark of *Warburgia ugandensis* collected in the Emariete Village Forest in the Monduli Juu district in Arusha Region of Northern Tanzania and identified by Mr. Emmanuel Mboya of the Tropical Pesticides Research Institute (Arusha, Tanzania). A ground root bark sample of *W. ugandensis* (Canellaceae) (200.6 g) was macerated in dichloromethane (3.5 L) over 4 days, and the obtained extract was dried in vacuo. The extract (5.3 g) was fractionated on a silica gel column (40 cm height × 4.08 cm diameter) and eluted with hexanes: Ethyl acetate (4:1), followed by ethyl acetate (2.9 L), and methanol (1.5 L), affording 13 fractions. Fraction 9 was further applied on a normal-phase silica gel (17.5 cm height × 1.7 cm diameter) eluted with hexanes: Ethyl acetate (4:1) and ethyl acetate, resulting in 10 fractions, WU-D-F9.1-10. Fraction Wu-D-F9.6 (2.3 mg) was identified as warburganal based on interpretation of NMR data and in comparison with the literature [34].

### 2.2. Estimation of the Amount of Compounds by ^1^H MNR:

Approximately 10 mg of each extract was dissolved in 0.6 mL of deuterated chloroform and analyzed on a Bruker AVANCE III 400 MHz NMR (Bruker, Billerica, MA, USA). The ^1^H NMR of *Cinnamosma* extracts were first measured to identify the signals of the solvents and abundant compounds. The most abundant compound was then selected, and each of its proton signals was integrated (and calibrated to 1 for 1 proton signal). The amounts of the other compounds in the extract relative to the most abundant compound was thus obtained by the integration of all the signals in the ^1^H NMR spectrum (except solvent). The percentage amount of the most abundant compound was estimated by using the following equation:

(1) Percentage of the major compound=(1−S) × 100,

where *S* = Sum of 1-proton signal integrations of the other identified compounds present in the ^1^H NMR.

### 2.3. Mosquito Colony

The present study utilized a colony of *Ae. aegypti* (Liverpool strain) derived from eggs, which were obtained from the MR4 as part of the BEI Resources Repository, National Institute of Allergy and Infectious Diseases (NIAID), National Institutes of Health (NIH) (LVP-IB12, MRA-735, deposited by M.Q. Benedict). Eggs were raised to adults, as described previously [35]. First instar larvae and adult females (5 to 10 days post-eclosion) were used for bioassays.

### 2.4. Toxicology Experiments

The larval toxicities of the extracts and isolated compounds were evaluated using an established assay [20,36,37]. In brief, 5 1st instar larvae were placed into each well of a 24-well Falcon Multiwell plate (Becton Dickinson Labware, Franklin Lakes, NJ) containing 985 μL of dH_2_O and 5 μL of a food solution (13 mg/mL of finely ground fish food flakes in dH_2_O; Tetramin, Blacksburg, VA). Ten microliters of an extract or compound (dissolved in 100% acetone) or 100% acetone (solvent control) were added to each well. In some experiments, 100% DMSO was used as the solvent (see Results). The plates were placed in a rearing chamber (28 °C, 80% relative humidity, 12:12 light: dark) for 24 h before larval toxicity was assessed. Larvae were considered dead if they did not move after prodding with a fine needle or pipette tip. Efficacy was defined as the percentage of larvae that died within 24 h after correcting for solvent effects using Abbot’s formula [38]. The screening concentrations used for the extracts (50 µg/mL) and isolated drimane sesquiterpenes (100 µM) were chosen based on preliminary scouting experiments with the bark extract and CDIAL (**1**), which resulted in 50–100% efficacy. In experiments testing for potential larvicidal synergy between CDIAL (**1**) and CMOS (**3**), the concentrations chosen (50 µM CDIAL, 75 µM CMOS) were estimates of the approximate molarity of each compound in the root extract at the screening concentration (50 µg/mL).

Adulticidal efficacy was evaluated using an established assay [20,36,39]. After immobilization on ice, groups of 10 adult female mosquitoes (5–10 days post-emergence) were topically treated with 500 nL of an extract or compound (dissolved in 100% acetone) or 100% acetone (solvent control) and transferred to small cages (16 oz. containers) with access to 10% sucrose. The compounds or solvent were delivered to the thorax of mosquitoes with a repeating dispenser (PB600-1, Hamilton, Reno, NV). The cages were provided with cotton wicks soaked in 10% sucrose and held in a rearing chamber (28 °C, 80% relative humidity, 12:12 light: dark) for 24 h before assessing toxicity. Efficacy was defined as the percentage of mosquitoes that were incapacitated (i.e., dead or unable to fly) within 24 h [20,36,39] after correcting for solvent effects using Abbott’s formula [38]. The screening doses used for the extracts (2.5 µg/mosquito) and isolated drimane sesquiterpenes (5 nmol/mosquito) were chosen based on preliminary scouting experiments, which resulted in 50–100% efficacy.

In some experiments, the compounds were injected directly into the hemolymph of the adult females using a pulled-glass capillary needle and Nanoject II injector (Drummond Scientific Company, Broomall, PA, USA). Each mosquito received an injection of 500 nL of phosphate-buffered saline (Thermo Scientific, Waltham, MA, USA) containing 3 mM of a compound (1.5 nmol/mosquito) or 3% DMSO (the solvent control). The efficacy was determined 24 h after injection, as described above.

### 2.5. Antifeedant Experiments

The antifeedant activity was assessed using a capillary feeding (CAFE) choice assay [20,40]. Adult female mosquitoes (5–10 days post-emergence) were starved for 24 h but provided with water-soaked cotton. Groups of 5 mosquitoes were transferred to *Drosophila* vials (28.5 × 95 mm; VWR International, Radnor, PA, USA) and covered with cotton plugs containing 2 holes to allow for the insertion of 5 μL calibrated glass capillaries (VWR International). The control capillary was filled with 5 μL of 10% sucrose containing 0.01% trypan blue (to provide contrast) and 1% acetone (the solvent). The treatment capillary was filled with 5 μL of 10% sucrose containing 0.01% trypan blue and an extract or isolated compound dissolved in 100% acetone. In some experiments, 100% DMSO was used as the solvent (see Results). The capillaries were capped with mineral oil to minimize evaporative losses. Vials with capillaries but no mosquitoes were also included to account for evaporative losses. The vials were held in a rearing chamber (28 °C, 80% relative humidity, 12:12 light: dark) for 18 to 20 h before measuring the volume of sucrose remaining in each capillary. After correcting for evaporation, the antifeedant index was calculated by subtracting the volume consumed from the treatment capillary from that of the control capillary and dividing by the total volume consumed from both capillaries [41]. The mean antifeedant indices were compared using GraphPad Prism (version 7) software with a one-way ANOVA and Tukey’s multiple comparisons test. The screening concentrations used for the extracts (50 µg/mL) and isolated drimane sesquiterpenes (1 mM) were chosen based on preliminary scouting experiments with the bark extract and CDIAL (**1**), which resulted in antifeedant indices of ~0.5.

### 2.6. Computational Docking

CDIAL (**1**) and CML (**4**) were docked to a potential binding pocket centered around Cys684 in a structural model of *An. gambiae* TRPA1 (AgTRPA1; AGAP004863) that was built and described previously [42] using a combination of homology and ab initio modeling approaches based on the human TRPA1 structure (PDB accession number 3J9P). A 96 × 68 × 78 grid box with a grid spacing of 0.375 Å centered around Cys684 defined the region of the protein that ligands would explore. Five hundred docking runs were performed for each ligand. All docking calculations were performed with the Lamarckian genetic algorithm using Autodock 4.2 [43].

## 3. Results

### 3.1. CDIAL and/or CMOS Are the Major Drimane Sesquiterpenes in the Bark, Roots, and Leaves

**Figure 2:**
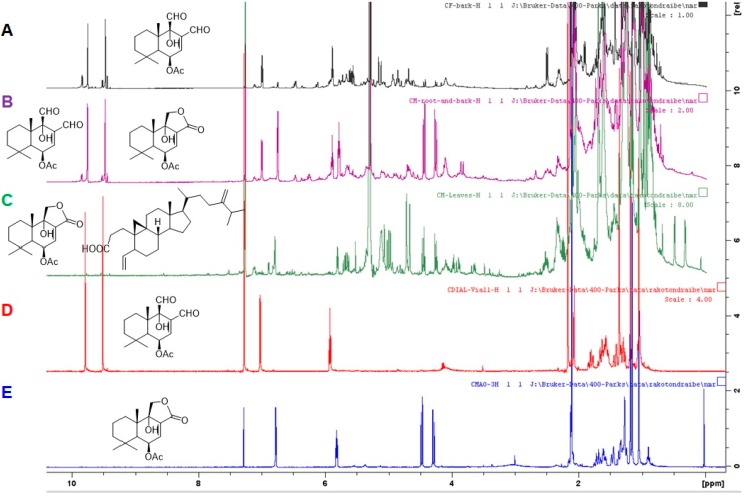
^1^H NMR spectra of *Cinnamosma* extracts, CDIAL (**1**), and CMOS (**3**). (**A**): Bark extract, (**B**): Root extract, (**C**): Leaf extract, (**D**): CDIAL and (**E**): CMOS.

The drimane-sesquiterpene content of the dichloromethane extracts of the bark, roots, and leaves of *Cinnamosma* spp. was profiled using one-dimensional ^1^H NMR (Figure 2A–C). Compounds in each extract were identified using our previously isolated compounds CDIAL (**1**) and CMOS (**3**) as reference spectra (Figure 2D,E, respectively). Results showed that the bark extract (Figure 2A) contained **1** as the major drimane constituent with a small amount of **3** (Table 1). CPCD (**7**), and POLYG (**9**) were also present, but in small amounts. The relative abundance of **1** in the bark extracts was ~60% of the total detectable/identifiable sesquiterpenes (Table 1). The ^1^H NMR spectrum of the root extract (Figure 2B) showed the presence of **1** and **3** in a 2:3 ratio, while the leaves (Figure 2C) showed a high amount of **3** (Table 1) and a small amount of a *seco*-triterpene derivative previously isolated from *C. fragrans* [44]. The leaves did not have detectable amounts of **1** (Table 1).

### 3.2. Relative Insecticidal Activities of Plant Extracts and Isolated Drimane Sesquiterpenes against Adult female and larval Mosquitoes

**Figure 3:**
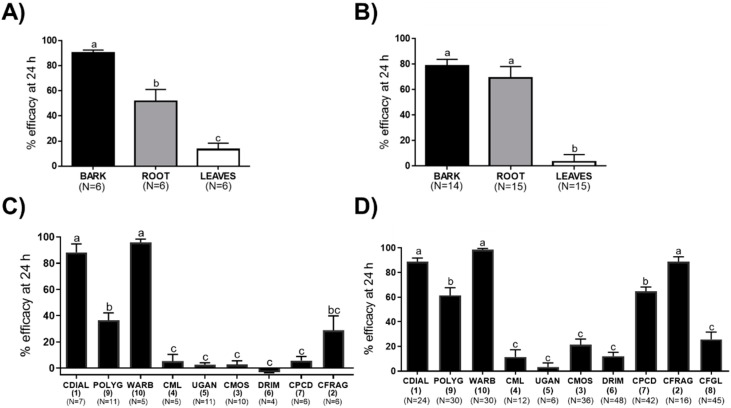
Insecticidal efficacy of plant extracts and isolated drimane sesquiterpenes against adult female (**A**,**C**) and larval (**B**,**D**) *Ae. aegypti* (Liverpool, LVP, strain). (**A**,**C**) Adulticidal efficacy was defined as the percentage of adults (after Abbott’s correction) that were incapacitated (dead or flightless) within 24 h when extract (2.5 µg/mosquito) or compounds (5 nmol/mosquito) were applied to the thoracic cuticle of the adult females. Values are means ± SEM; N = number of independent replicates of 10 females each. (**B**,**D**) Larvicidal efficacy in 1st instar larvae was defined as the percentage (after Abbott’s correction) that died within 24 h when extract (50 µg/mL) or compounds (100 µM) were added to the rearing water (100 µM). Values are means ± SEM; N = number of independent replicates of 5 larvae each. In all panels, lower-case letters indicate statistical categorization of the means as determined by a one-way ANOVA and Tukey’s multiple comparisons test (*p* < 0.05).

First, the insecticidal activities of the bark, root, and leaf extracts of *Cinnamosma* plants against *Ae. aegypti* (Liverpool, LVP, strain) was compared. As shown in Figure 3A, at the screening concentration (2.5 µg/mosquito), the bark extract had the strongest adulticidal efficacy within 24 h (90%) followed by the root (52%) and leaf (13%) extracts. As shown in Figure 3B, at the screening concentration (50 µg/mL), the bark and root extracts each had strong larvicidal efficacies within 24 h (~75%) that were significantly more efficacious than the leaf extract (~0.9%).

Next, the drimane sesquiterpenes isolated from the bark and root extracts were screened to determine which compounds contributed to their adulticidal and larvicidal activities. In addition, we tested WARB (**10**) isolated from *W. ugandensis*. In adult females (Figure 3C), at the screening dose (5 nmol/mosquito), WARB (**10**) and CDIAL (**1**) exhibited the strongest efficacy, whereas POLYG (**9**), CFRAG (**2**), and CFGL (**8**) were moderate to weakly toxic; CML (**4**), UGAN (**5**), CMOS (**3**), DRIM (**6**), and CPCD (**7**) were nominally toxic (Figure 3C). To determine if the significantly lower topical adulticidal activity of **9** relative to **1** was due to weaker cuticular penetration, we injected each into the hemolymph of the adult females (1.5 nmol/mosquito). The toxic efficacy of **9** (49.86% ± 4.84%; N = 7) within 24 h was significantly lower (unpaired *t*-test; *p* < 0.05) than that of **1** (84.19% ± 6.34%; N = 16) when delivered by injection.

In larvae (Figure 3D), at the screening concentration (100 µM), WARB (**10**), CDIAL (**1**), and CFRAG (**2**) were among the most toxic, while POLYG (**9**) and CPCD (**7**) were moderately efficacious; CML (**4**), UGAN (**5**), CMOS (**3**), DRIM (**6**), and CFGL (**8**) were among the least toxic. Previously, we found that **7** dissociated into monomers of **1** in DMSO [45]. Thus, in a parallel experiment, we directly compared the larvicidal efficacy of **7** when dissolved as a stock solution in acetone vs. DMSO. The 24 h larvicidal efficacy of **7** from a DMSO stock (100% ± 0.0%; N = 6) was significantly greater (*p* <0.01, unpaired *t*-test) than that from an acetone stock (55.5% ± 11.1%; N = 6).

**Figure 4:**
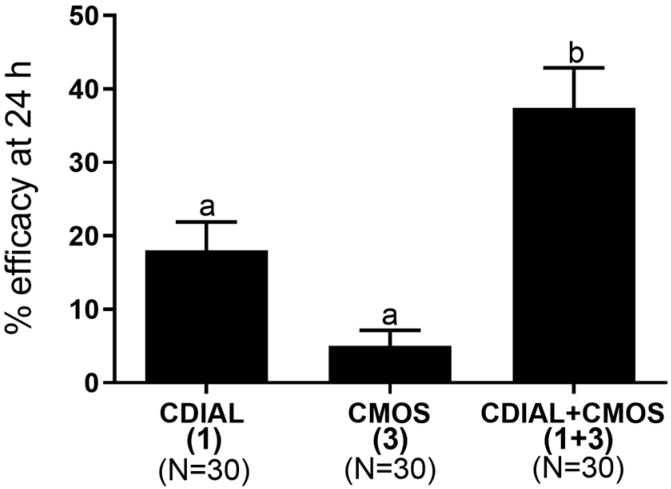
Synergism between CDIAL (**1**) and CMOS (**3**) against 1st instar larvae of *Aedes aegypti*. Larvicidal efficacy in 1st instar larvae was defined as the percentage (after Abbott’s correction) that died within 24 h after adding CDIAL (50 μM), CMOS (75 µM), or the combination of CDIAL (50 μM) and CMOS (75 µM) to the rearing water. Values are means ± SEM; N = number of independent replicates of 5 larvae each.

Given that the root extract exhibited similar larvicidal activity as the bark extract (despite a lower abundance of **1**), we tested for potential synergy between **1** and **3**; the latter is highly abundant in the root extract (Table 1). When tested individually, 50 µM CDIAL (**1**) or 75 µM CMOS (**3**) resulted in 18% or 5% larvicidal efficacy within 24 h, respectively (Figure 4). When tested together at these concentrations, they resulted in 37% larvicidal efficacy (Figure 4), which was significantly greater than CDIAL and ~1.5-times larger than expected based on the sum of their individual activities (~23%).

### 3.3. Relative Antifeedant Activity of Plant Extracts and Isolated Drimane Sesquiterpenes against Adult Female Mosquitoes

**Figure 5:**
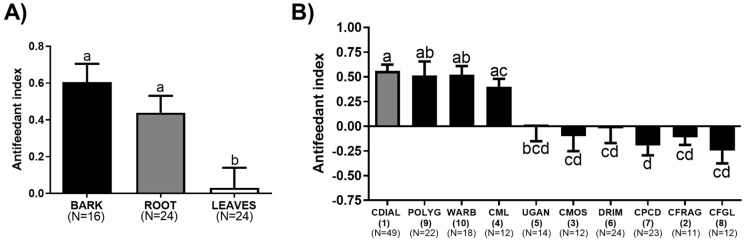
The antifeedant activity of plant extracts (**A**) and isolated drimane sesquiterpenes (**B**) in choice capillary feeding (CAFE) assays with adult female *Ae. aegypti* (LVP strain) mosquitoes. At the time of feeding, each group of five mosquitoes was offered two glass capillaries filled with 10% sucrose and 0.01% trypan blue. The control capillary included 1% acetone (the solvent), and the treatment capillary included 1% acetone and an extract (50 μg/mL) or a drimane sesquiterpene (1 mM). The difference in volume consumed between the capillaries was used to calculate the antifeedant activity. See Figures S1 and S2 for consumption volumes. Values are means ± SEM; N = number of independent replicates of five mosquitoes each. Lower-case letters indicate statistical categorization of the means as determined by a one-way ANOVA and Tukey’s multiple comparisons test (*p* < 0.05).

To assess the relative antifeedant activity of the bark, root, and leaf extracts against adult female *Ae. aegypti* (LVP strain), we used a capillary feeding (CAFE) choice bioassay [20,40,46]. In brief, this assay compared the consumption of 10% sucrose by mosquitoes from two glass capillaries over an 18 to 20 h period: A control capillary was treated with the solvent (1% acetone or DMSO), and a treatment capillary was treated with an extract or isolated compound. Mosquitoes consumed significantly less sucrose from the capillaries treated with 50 µg/mL bark or root extract vs. control capillaries (Figure S1), indicative of antifeedant activity. On the other hand, mosquitoes consumed similar volumes of sucrose from the capillaries treated with the leaf extract vs. the control capillaries (Figure S1), indicative of nominal antifeedant activity. As demonstrated in Figure 5A, the antifeedant indices of the bark and root extracts were similar to each other and significantly greater than that of the leaf extract.

To determine which compounds contributed to the antifeedant activity, we performed similar assays with the compounds isolated from the bark and roots. In addition, we tested WARB (**10**) isolated from the bark of *W. ugandensis*. Mosquitoes consumed significantly less sucrose from the capillaries treated with 1 mM POLYG (**9**), WARB (**10**), CDIAL (**1**), or CML (**4**) vs. control capillaries (Figure S2), indicative of antifeedant activity. Mosquitoes consumed similar volumes of sucrose from capillaries treated with 1 mM UGAN (**5**), CMOS (**3**), DRIM (**6**), CPCD (**7**), CFRAG (**2**) or CFGL (**8**) vs. control capillaries, indicative of nominal antifeedant activity (Figure S2). As shown in Figure 5B, the antifeedant efficacies of CDIAL (**1**), POLYG (**9**), WARB (**10**), and CML (**4**) were similar among one another and significantly greater than the other compounds.

In a parallel experiment, we directly compared the antifeedant efficacy of 250 µM CPCD (**7**) when dissolved as a stock solution in acetone vs. DMSO. When using acetone as the solvent, mosquitoes consumed similar volumes of sucrose from capillaries treated with **7** vs. the control capillaries (paired *t*-test; *p* = 0.98). However, when using DMSO as the solvent, mosquitoes consumed significantly less sucrose from the capillaries treated with **7** vs. the control capillaries (paired *t*-test; *p* < 0.05). As such, the antifeedant index of **7** from a DMSO stock (0.20 ± 0.07; N = 10) was significantly greater (*p* < 0.01, unpaired *t*-test) than that of an acetone stock (−0.02 ± 0.07; N = 10).

### 3.4. Computational Docking of CML and CDIAL to AgTRPA1

**Figure 6:**
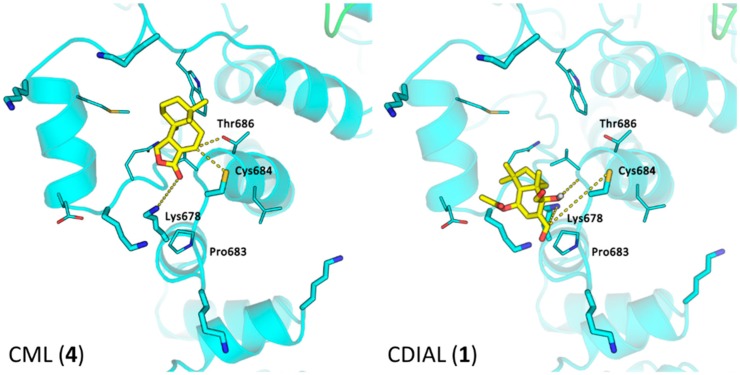
Structural models of CML (**4**) or CDIAL (**1**) in the putative CDIAL-binding site of AgTRPA1. Potential interactions between ligand (yellow) and residues of AgTRPA1 (cyan) as predicted by computational docking are shown. Several nearby residues are labeled and shown in licorice representation.

To test whether CML (**4**) can potentially bind mosquito TRPA1 channels in a similar manner to CDIAL (**1**), we computationally docked both to the putative binding pocket centered around Cys684 in AgTRPA1 that was identified and described previously [42]. The docking results for **1** revealed that both the C-12 and C-11 aldehyde groups were located near Lys678, which makes these sites readily accessible to attack by the lysine’s amino group to form a covalent adduct (Figure 6). In contrast, **4** adopts a different binding pose from **1** (Figure 6) and binds AgTRPA1 less effectively than **1** as suggested by their binding scores (**4** = −4.9 kcal/mol; **1** = −7.2 kcal/mol). Additionally, although the lactone moiety of **4** stays close to Lys678 and forms a hydrogen bond with the lysine’s amino group, the lactone carbonyl group was less electrophilic than the aldehydes, and its orientation makes it less reactive towards the lysine amino group.

## 4. Discussion

The present study was the first to compare the insecticidal and antifeedant activities of dichloromethane extracts from different parts of the *Cinnamosma* plants and drimane sesquiterpenes isolated from these extracts against mosquitoes. Our results advance our understanding of the potential use of these plants as sources of natural insecticides and repellents for mosquito control, and the chemical features of drimane sesquiterpenes that contribute to their insecticidal and/or antifeedant bioactivities against mosquitoes.

### 4.1. Insecticidal Activity of Plant Extracts and Isolated Compounds

The acute topical toxicity experiments against adult female *Ae. aegypti* revealed that bark and root extracts of *Cinnamosma* spp. were significantly more toxic than leaf extracts (Figure 3A). This trend extends to insecticidal efficacy against first instar larvae, where it was found that the bark and root extracts were stronger insecticides than the leaf extracts from these plants (Figure 3B). Our results are similar to those reported by Pavela et al. [21], who found that essential oil from the bark of *C. madagascariensis* was more toxic to larval *Culex quinquefasciatus* compared to essential oil from the leaves. Thus, the bark and roots of *Cinnamosma* sp. appear to be the major sites of insecticidal compound production. However, this trend does not apply to all plants, because in some species, leaves have been known to produce insecticidal compounds (e.g., [23,47,48]).

The relative insecticidal activities of the bark, root, and leaf extracts can largely be explained by differences in the relative concentration of CDIAL (**1**) within the extracts. In the bark, **1** was highly abundant and composed ~60% of the total sesquiterpenes. On the other hand, **1** only composed ~30% of the total sesquiterpenes in the root extract and was not detectable in the leaf extract. As such, the adulticidal efficacy of the root extract was ~50% lower than the bark extract, and the leaf extract was nominally efficacious (Figure 3A). Previously, we demonstrated that **1** was a superior adulticidal compound against *Ae. aegypti* compared to CFRAG (**2**) and CMOS (**3**) [20]. In the present study, **1** was the only compound isolated from the bark and root extracts of *Cinnamosma* to elicit >50% efficacy in adult females at the screening dose (Figure 3D; note WARB was isolated from *W. ugandensis*). Thus, our results suggest that CDIAL is the principal active component of the *Cinnamosma* bark and root extracts responsible for their adulticidal activities.

In contrast, the larvicidal activity of the root extract is more complex. Notably, despite the lower content of **1** in the root extract, its larvicidal efficacy was surprisingly similar to that of the bark extract within 24 h. One possible explanation for this result is synergism of **1** with another abundant compound in the root extract, such as **3**. Remarkably, when added to the larval rearing water in a similar molar ratio as found in the root extract, we found that the efficacy of **1** and **3** were ~1.5-times greater than the summation of their individual efficacies, suggesting synergy between the two compounds. It is unclear how **3** enhances the efficacy of **1**, but it has been found that some essential oils of plants are more active than the isolated major compounds against larval *Ae. aegypti* and that some essential oils are able to synergize the toxicity of established insecticides by inhibiting detoxification mechanisms, such as cytochrome P450s and glutathione S-transferases [49,50,51,52]. Thus, one hypothesis to test in future studies is that **3** enhances the insecticidal activity of **1** by inhibiting detoxification systems in a similar fashion as essential oils.

Another potential, and not mutually exclusive, explanation for the greater than expected larvicidal efficacy of the root extract is that other bioactive components in the root extract were toxic to larvae. Consistent with this notion, **1** was not the only *Cinnamosma*-derived compound to exhibit strong larvicidal efficacy within 24 h at the screening concentration. **2** was similarly larvicidal to **1**, confirming our earlier study [20]. In addition, POLYG (**9**) and CPCD (**7**) exhibited over 50% efficacy at the screening concentration. Thus, in addition to **1**, other aldehyde-bearing drimane sesquiterpenes in the root extract, such as **9**, **7**, and **2,** may contribute to the greater than expected larval toxicity, especially if their toxicity is also synergized by **3**. However, the abundances of these other active compounds in the root extract are very low compared to **1** (Figure 2).

Previously, we demonstrated that the weakly-bioactive dimer **7** dissociated into highly bioactive monomers of **1,** and the presence of DMSO enhanced this dissociation [45]. Consistent with the previous study, we found that **7** was significantly more larvicidal when its stock solution was prepared in DMSO vs. acetone, suggesting **7** is a pro-insecticide that requires dissociation into **1** before becoming toxic. This phenomenon may explain why **7** prepared in 100% acetone was relatively non-toxic when applied topically to the hydrophobic cuticle of adult females. That is, on the cuticle, **7** would not have an opportunity to dissociate into **1** unless it was rapidly absorbed into the aqueous hemolymph. Compared to **7**, dimer **2** is more stable and does not dissociate into monomers [30]. Thus, its prominent larvicidal activity suggests it is bioactive in its dimeric form. Consistent with this notion, we have previously observed that **2** acutely activates mosquito TRPA1 channels expressed heterologously in *Xenopus* oocytes [20].

In addition to direct toxic effects of the drimane sesquiterpenes on larval mosquitoes, we cannot rule out that some of the compounds are indirectly toxic to larvae by influencing the microbial composition in their rearing water and/or guts. Previous studies on *Ae. aegypti* have shown that the gut microbiota, which they acquire from their rearing water, strongly influence mosquito development and survival [53,54]. Moreover, essential oils of *C. fragrans* and some sesquiterpenes have antibiotic properties [55,56,57,58,59]. Thus, future studies should explore whether **1** and other drimane sesquiterpenes in the bark and root extracts influence the microbial composition of the rearing water and/or gut in a manner that would cause acute larval toxicity or perturb development.

### 4.2. Insights into the Insecticidal SAR of CDIAL-Like Drimane Sesquiterpenes

Comparing the structures and relative insecticidal activities of the monomeric drimane sesquiterpenes allow for valuable insights into the insecticidal SAR of these molecules. First of all, only the aldehyde-bearing monomers (**1**, **9**, **10**) were insecticidal, whereas all of the lactone-bearing monomers (**3**–**6**) were nominally toxic. Notably, **3** and **4** are identical to **1** and **9**, respectively, with the exception of the lactone functions replacing the aldehydes (Figure 1). Thus, the presence of highly reactive aldehydes appears to be essential for the larvicidal and adulticidal bioactivities of these molecules.

Comparing the structures of the insecticidal monomers (**1**, **9**, **10**), which are nearly identical with the exception of a hydroxyl (-OH) on C-9 in **1** and **10**, and an acetyl (-OAc) on C-6 in **1** (Figure 1), reveals that the -OH plays a key role in modulating insecticidal activity. That is, the insecticidal efficacies of **1** and **10**, which possess the -OH are 1–3 times greater than **9**. Given that **9** was still less efficacious than **1** when injected into the hemolymph, the -OH group does not appear to enhance cuticular penetration. Instead, the -OH group likely enhances the toxicity of the molecule to mosquitoes by enhancing interactions with its molecular target(s) and/or making it a poorer substrate for mosquito detoxification systems (e.g., cytochrome P450s). Future studies on the mode of **1**’s insecticidal action and how it is detoxified by mosquitoes will be required to fully elucidate how the -OH group modulates insecticidal activity. On the other hand, the -OAc on C-6 does not appear to enhance or detract from the insecticidal efficacy against larvae or adults given the similar activities of **1** and **10**.

### 4.3. Antifeedant Activity of Plant Extracts and Isolated Drimane Sesquiterpenes

Similar to the trend found for insecticidal activity, the antifeedant efficacies of the bark and root extracts were significantly stronger than that of the leaf extract. Thus, the bark and roots of *Cinnamosma* species also appear to be the major sites of antifeedant compound production. In other plants, the leaves also produce antifeedant compounds [23,60,61].

Similar to the relative larvicidal activity of the extracts, the relative antifeedant efficacies of the extracts cannot completely be explained by differences in the relative concentrations of **1** within the extracts. Previously, and consistent with our current results, we have shown that **1** is a more effective antifeedant than **2** and **3** and is likely the principal antifeedant component of the bark extract [20]. However, the root extract produced a similar antifeedant effect as the bark extract despite a ~50% lower concentration of **1**. Thus, other compounds likely contribute to the antifeedant activity of the root extract. Consistent with this notion, **9** and **4** exhibited comparable antifeedant efficacy to **1**. However, given the low abundances of these compounds compared to **1**, we also cannot rule out potential synergistic effects of the antifeedant compounds with each other and/or the less active lactones and dimers.

In the present study, the dimer **7** was not antifeedant when prepared as a stock solution in 100% acetone, suggesting the compound does not substantially dissociate into monomers of **1** in the 10% sucrose solution over the 18 h course of the experiments or acutely alter feeding behavior after ingestion by the mosquito. However, when prepared as a stock solution in DMSO, the antifeedant activity of **7** was unmasked, suggesting that dissociation into **1** was necessary for bioactivity. These results are consistent with those from the aforementioned larval toxicity experiments that demonstrated DMSO enhanced the larvicidal activity of **7**.

### 4.4. Insights into Antifeedant SAR of CDIAL-Like Drimane Sesquiterpenes

Comparing the structures and relative antifeedant activities of the monomeric drimane sesquiterpenes allow for valuable insights into the antifeedant SAR of these molecules. Notably, all of the aldehyde-bearing compounds (**1**, **9**, **10**) were antifeedant to mosquitoes, whereas all of the lactone-bearing compounds, except for **4**, were nominally antifeedant. We have previously demonstrated that the relative antifeedant activities of **1**, **2**, and **3** were correlated with their relative agonism of mosquito TRPA1 channels and that TRPA1 was essential for the antifeedant activity of **1** [20]. The strong antifeedant activity of **9** and **10** is likely due to agonism of TRPA1 channels because both are potent agonists of vertebrate TRPA1 channels [62,63] and known antifeedants against a wide variety of insects [64,65,66,67,68]. The electrophilic aldehyde groups of **1**, **9**, and **10** are likely critical for interacting with nucleophilic cysteine and lysine residues in the NH_2_-terminal domain of TRPA1 channels [20,69,70,71]. The similar antifeedant efficacies of these compounds suggest that the -OH on C-9 (**1**, **10**) and the -OAc on C-6 (**1**) do not substantially modulate the antifeedant activity and by extension, the agonism of mosquito TRPA1. In contrast, as mentioned above, the presence of the -OH on C-9 enhanced insecticidal activity. These divergent results are consistent with the idea that **1** has distinct mechanisms of insecticidal and antifeedant action [20].

Whereas agonism of TRPA1 likely explains the strong antifeedant activities of **1**, **9**, and **10**, the antifeedant activity of **4** is puzzling. **4** is a natural lactone derivative of **9** wherein the electrophilic aldehydes are replaced with a relatively less reactive lactone function (Figure 1). This substitution would be expected to make **4** an inferior agonist of TRPA1 channels and antifeedant, as we have previously observed for **3**, the lactone derivative of **1** [20]. Consistent with this notion, computational docking simulations of **4** with AgTRPA1 revealed that although it has the potential to fit in the pocket near Cys684, its binding pose/strength and reactivity suggest it is a weaker agonist compared to **1** (Figure 6). Furthermore, CML does not induce detectable agonism of heterologously-expressed TRPA1 channels from vertebrates [63]. However, the antifeedant efficacy of **4** was similar to **1**, **9**, and **10**. Moreover, in the hemipteran pests *Bemisia tabaci* and *Myzus persicae*, **4** was antifeedant, albeit with apparent less potency than **9** [72]. Thus, our results suggest that the mechanism of **4**’s antifeedant activity is distinct from **1**, **9**, and **10**, and remains to be elucidated. In humans, **4** has been shown to interact with acetylcholine receptors [73], and possess cytotoxic activity [44]. Whether **4** can interact with similar receptors or have similar activities in insects to induce antifeedant or repellent behaviors remains to be determined.

## 5. Conclusions

In conclusion, the present investigation reveals the insecticidal and antifeedant properties of the bark and root extracts of *Cinnamosma* sp. and their major drimane sesquiterpenes against *Ae. aegypti* larvae and adults. Further elucidation of the structure–activity relationships of these molecules, their potential synergies, and modes/mechanisms of action will facilitate the development and formulation of next-generation insecticides and repellents for mosquito control.

## Figures and Tables

**Table 1:** Relative abundances of compounds **1** and **3** in the extracts of *Cinnamosma* species. Percentages were estimated using the integration of the ^1^H signals.

Plant Extract	Relative Abundance of Compound	
	CDIAL **(1)**	CMOS **(3)**
**Bark**	~60%	<5%
**Root**	~30%	~45%
**Leaves**		~30%

## Supplementary Materials

The following are available online at https://www.mdpi.com/2075-4450/10/11/373/s1, Figure S1. Volumes of sucrose consumed in control and treatment capillaries during choice CAFE assays in adult female *Ae. aegypti* (LVP strain); Figure S2. Volumes of sucrose consumed in control and treatment capillaries during choice CAFE assays in adult female *Ae. aegypti* (LVP strain).
