# Supplementary material for: Insecticidal and Antifeedant Activities of Malagasy Medicinal Plant (Cinnamosma sp.) Extracts and Drimane-Type Sesquiterpenes against Aedes aegypti Mosquitoes
Source: Insects. 2019 Oct 25;10(11):373. doi: 10.3390/insects10110373 (PMC6920793; doi:10.3390/insects10110373)
Supplement: Supplementary file 1 [file insects-10-00373-s001.zip › Fig. S2.pdf]

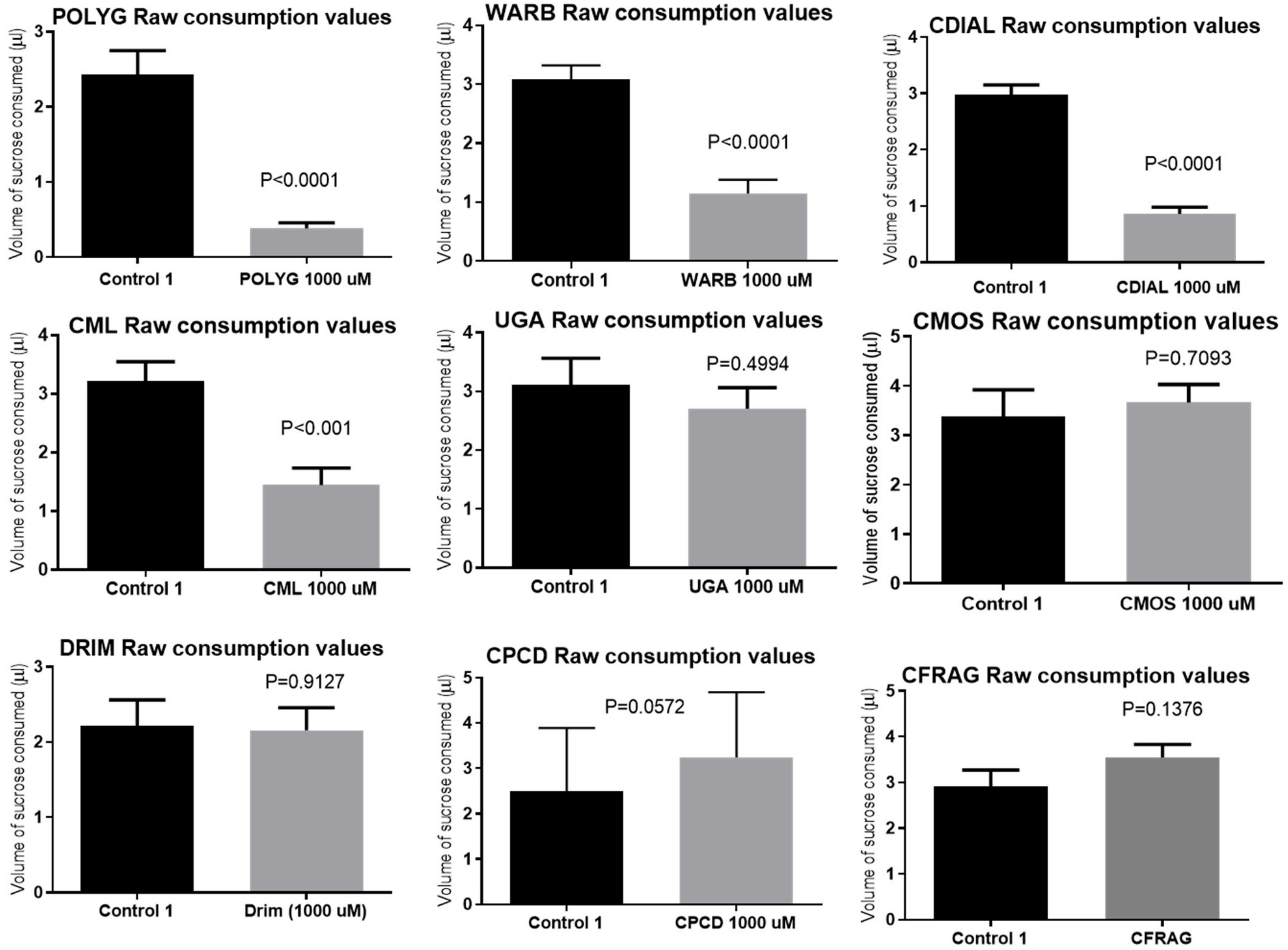

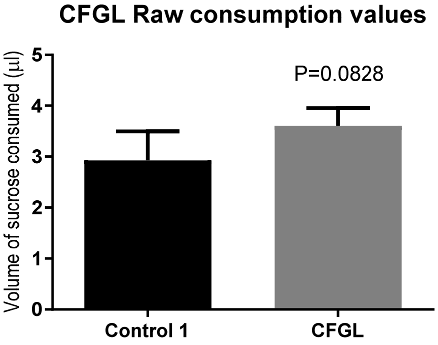

Fig. S2. Volumes of sucrose consumed in control and treatment capillaries during choice CAFE assays in adult female *Ae. aegypti* (LVP strain). The compound (1 mM) used in the treatment capillaries is indicated. As described in the methods, the differences in volume consumed between the capillaries were used to calculate the antifeedant indices in Fig. 6A. Values are means ± SEM; N= 10-24 independent replicates of 5 mosquitoes each. Differences in volumes consumed between control and treatment capillaries were determined by paired t-tests ($P < 0.05$).
